# Course and outcome of patients with alcohol use disorders following an alcohol intervention during hospital attendance: mixed method study

**DOI:** 10.1192/bjo.2020.138

**Published:** 2020-12-02

**Authors:** Sophia E. Chambers, David S. Baldwin, Julia M. A. Sinclair

**Affiliations:** Clinical and Experimental Sciences, Faculty of Medicine, University of Southampton, UK; Clinical and Experimental Sciences, Faculty of Medicine, University of Southampton, UK; and University Department of Psychiatry and Mental Health, University of Cape Town, South Africa; Clinical and Experimental Sciences, Faculty of Medicine, University of Southampton, UK

**Keywords:** Alcohol use disorder, mixed methods, outcome studies, cohort

## Abstract

**Background:**

Alcohol-related presentations to acute hospitals in the UK are increasing, but little is known of the clinical characteristics or natural history of this patient group.

**Aims:**

To describe the clinical characteristics, drinking profile and trajectory of a cohort of patients with alcohol use disorder (AUD) attending hospital, and explore participant perspectives of the impact of hospital attendance on their relationship with alcohol.

**Method:**

We conducted a mixed method, prospective, observational cohort study of patients with AUD seen in an acute hospital. Participants were interviewed with a range of questionnaires at baseline and followed up on at 6 months. A subsample also completed in-depth qualitative interviews.

**Results:**

We recruited 141 patients; 132 (93.6%) were followed up at 6 months and 26 completed qualitative interviews. Of the 141 patients, 60 (42.6%) stated the index hospital episode included the first discussion of their alcohol use in a secondary care setting. Most rated discussion of their alcohol use in hospital as ‘very positive’ or ‘positive’ (102/141, 72.3%), but lack of coordinated care with community services undermined efforts to sustain change. At 6 months, 11 (7.8%) patients had died, but in those who survived and completed assessment (*n* = 121), significant and clinically meaningful improvements were seen across a range of outcomes, with 55 patients (45.5%) showing a favourable drinking outcome at 6 months.

**Conclusions:**

Patients with AUD have high levels of morbidity and mortality, yet many made substantial changes following intervention in hospital for their alcohol use. Prospective trials need to identify the effect of alcohol care teams in optimising this ‘teachable moment’ for patients.

The physical and psychological complication arising from excessive alcohol consumption accounts for 5.1% of disease burden, and 5.3% of deaths globally.^1^ Recent data show that presentations of patients with alcohol-related conditions to acute hospitals are increasing in England,^2^ with a pooled prevalence estimated within the UK hospital system of 19.8% for ‘harmful drinking’ and 10.3% for alcohol dependence.^3^

## Managing alcohol related harms

Brief interventions have been shown to be effective for people drinking above lower risk levels.^4^ More structured longer-term treatment is often necessary for those who are severely dependent, who often also have additional complex health and social care needs,^5^ which place a significant burden on health systems.^6^ Between these groups are a large number of people who would also be defined as having an alcohol use disorder (AUD),^7^ but for whom there is very little evidence on the natural history of the disorder, or the most effective interventions.

Given the disparity in numbers between those meeting criteria for AUD and those accessing specialist treatment,^8,9^ hospital attendance may present an ideal ‘teachable moment’ to engage individuals across the spectrum of AUD in discussions about their drinking.

## Development of alcohol care teams (ACTs)

The National Health Service (NHS) 10-year plan^10^ aims to develop optimised Alcohol Care Teams within hospitals as part of reducing health inequalities. The structure and competencies required to deliver these services have been defined,^11^ and previous findings suggest alcohol interventions delivered in hospital might be effective,^12–14^ and received positively by patients.^15,16^ However, there is limited evidence about the clinical characteristics of the patients seen, and what may constitute an effective intervention to engage people in behavioural change. Therefore, the aims of this study were to (a) describe the personal characteristics and drinking profile of a cohort of patients with AUD presenting to an acute hospital; (b) observe participant outcomes over 6 months following a hospital episode and (c) understand how participants perceive the impact of attending hospital, and receiving an alcohol intervention, had on their relationship with alcohol.

## Method

### Study design

**Fig. 1:**
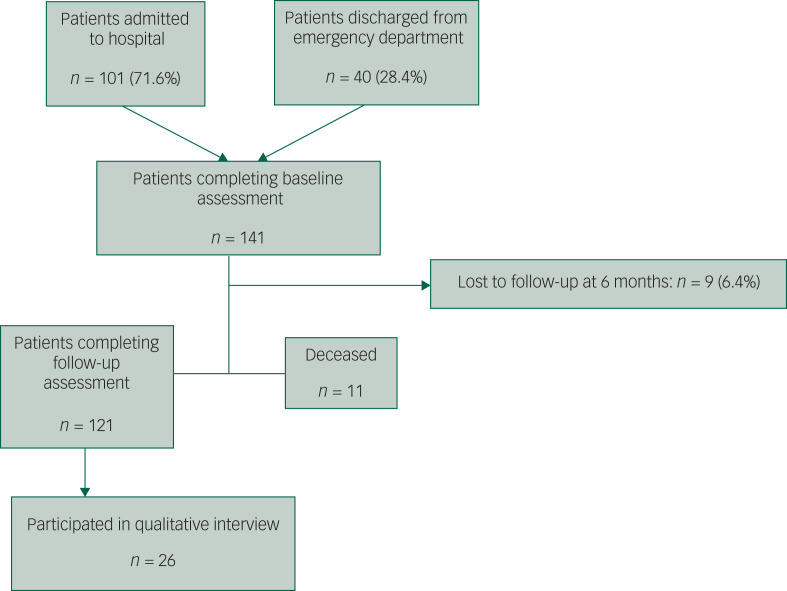
Flow of patients through the study.

This was a two-phase, mixed method study: an observational 6-month follow-up of individuals who had received an alcohol intervention during their hospital episode; and qualitative interviews with a subsample of participants to explore their experiences of problematic alcohol use and index hospital episode in greater detail (Fig. 1).

### Participants

Potentially eligible individuals were those either admitted to University Hospital Southampton, or who attended its Emergency Department, between September 2016 and March 2017; and who were identified as having an AUD and received any form of alcohol intervention (e.g. brief intervention, medically assisted withdrawal, referral to community services). Patients admitted for any presenting complaint were eligible. Exclusion criteria comprised a score of <8 on the consumption questions of the Alcohol Use Disorder Identification Test (AUDIT-C),^17^ age <18 years, inability to speak English and inability to provide informed written consent.

### Materials and procedures

Following their assessment and intervention (which included feedback to the patient, liaison with medical teams and onward referral where required), clinical staff from one of three teams (a pan-hospital consultant-led Alcohol Care Team, a ‘Vulnerable Adult Support Team’ based within the emergency department and the liaison psychiatry team) referred patients to the research team. After providing written consent, participants were interviewed during their hospital stay, or within 48 h after discharge, and again 6 months later (within 2 weeks either side of this date). Participants were reimbursed (£10) for their time, plus any travel costs, after completing the follow-up interview. Death certificates of individuals who were confirmed as deceased at the 6-month point were requested via the UK General Register Office website (https://www.gov.uk/order-copy-birth-death-marriage-certificate).

At baseline, sociodemographic characteristics, alcohol consumption over the past 6 months, level of dependence, anxiety and depression symptoms, readiness to change and any current or previous treatment for alcohol use were assessed, using a range of questionnaires (see Table 1); the same measures were readministered at 6 months. Details of the hospital episode, including length of stay and reason for attendance or admission, were collected retrospectively from medical notes.

**Table 1:** Description of questionnaires administered at baseline and follow-up

Questionnaire measure	Description	Score range
Participant information	Purpose-designed questionnaire capturing sociodemographic information, current/recent use of alcohol treatment services, and details of current hospital admission	Not applicable
7-day Timeline Follow-Back (TLFB)^18^	A self-report drinking assessment method to obtain estimates of daily drinking over the previous 7 days	Used to calculate total number of weekly units and number of heavy drinking days
Alcohol Use Disorder Identification Test (AUDIT)^19,20^	WHO screening tool for alcohol use disorders	0–40 (higher scores indicate more severe AUD) 20+ indicates probable dependence
Alcohol Use Disorder Identification Test – Consumption questions (AUDIT-C)^17^	First three questions of the AUDIT asking about level of alcohol consumption; a shortened screening test, often used in hospital settings	0–12 (higher scores indicate greater alcohol consumption)
Leeds Dependence Questionnaire (LDQ)^21^	A measure of psychological dependence on alcohol which can be used during periods of drinking or abstinence	0–30 (higher scores indicate greater dependence)
Problem Perception (scale derived from SOCRATES)^22^	A measure of problem awareness and recognition of the need to access help to change drinking	10–50 (higher scores indicate greater problem perception)
Taking Action (scale derived from SOCRATES)^22^	Assesses the steps the individual has taken, or is currently taking, toward addressing their drinking	6–30 (higher scores indicate greater levels of action)
Hospital Anxiety and Depression Scale (HADS)^23^	Measure of psychological distress	0–42 (higher scores indicate greater distress)

WHO, World Health Organization; SOCRATES, Stages of Change Readiness and Treatment Eagerness Scale.

A ‘positive’ outcome at 6-month follow-up was defined in two ways. First, as a favourable drinking outcome at 6 months, defined as zero heavy drinking days in the past week. European Medicines Agency^24^ guidelines define a heavy drinking day as >40 g of alcohol (5 UK units) for women, and >60 g of alcohol (7.5 UK units) for men. Second, as a clinically significant and reliable change in levels of psychological dependence (derived from scores on the Leeds Dependence Questionnaire (LDQ),^21^ as per Jacobson and Truax's^25^ guidelines). The method uses normative data to derive a cut-off value that indicates whether a follow-up score has moved closer to that of a ‘well-functioning’ population; norms for the LDQ are reported by Raistrick et al^26^ (see also www.result4addiction.net/ldq).

Qualitative interviews were conducted as part of a wider grounded theory study exploring personal experiences of problematic alcohol use and recovery.^27^ In-depth face-to-face interviews were held with 26 individuals who had completed both baseline and follow-up interviews. Participants were purposively sampled to reflect the range of characteristics (e.g. demographics, drinking profile) found within the overall cohort; their characteristics are presented alongside those of the whole cohort to allow for comparison (see Table 2). A topic guide provided structure for qualitative interviews, and participants were encouraged to talk freely about their use of alcohol and experience of attending hospital. Participants’ own data from the quantitative analysis was brought into the interview to stimulate discussion of the possible impact of the hospital episode on drinking behaviour and other measured variables. Interviews lasted 40–181 min (mean 105 min) and were audio-recorded, with consent.

**Table 2:** Participant characteristics at baseline including personal characteristics, drinking profile, use of alcohol treatment services, details of hospital episode and self-reported psychological distress

Characteristic	Whole cohort, *n* = 141	Qualitative sample, *n* = 26
Sociodemographics		
Male, *n* (%)	100 (70.9)	19 (73.1)
Age, mean (s.d.)	50.8 (13.7)	52.4 (11.3)
White, *n* (%)	134 (95.0)	26 (100.0)
In a relationship, *n* (%)	34 (24.1)	5 (19.2)
Living with others, *n* (%)	59 (41.8)	11 (42.3)
Currently employed or in education, *n* (%)	30 (21.3)	6 (23.1)
Current smoker, *n* (%)	88 (62.4)	17 (65.4)
Current substance use, *n* (%)	23 (16.3)	5 (19.2)
Drinking profile and use of alcohol services		
Alcohol units consumed in the week before admission, mean (s.d.)	140.6 (121.0)	188.3 (146.1)
Number of heavy drinking days^a^ in the week before admission, median (IQR)	7 (3–7)	7 (5.75–7)
AUDIT score, mean (s.d.)	29.7 (7.5)	30.9 (7.0)
AUDIT-C score, mean (s.d.)	11.0 (1.8)	11.1 (1.7)
LDQ score, mean (s.d.)	18.4 (10.3)	20.9 (9.1)
Problem Perception score, mean (s.d.)	37.5 (9.2)	40.7 (6.8)
Taking Action score, mean (s.d.)	22.0 (6.7)	22.3 (7.7)
Alcoholics Anonymous attendance, *n* (%)		
Past month	9 (6.4)	2 (7.7)
1 to <6 months	7 (5.0)	3 (11.5)
6–12 months	3 (2.1)	2 (7.7)
≥12 months	28 (19.9)	6 (23.1)
Never	94 (66.7)	13 (50.0)
Specialist alcohol treatment service attendance, *n* (%)^b^		
Past month	20 (14.2)	6 (23.1)
1 to <6 months	13 (9.2)	4 (15.4)
6–12 months	6 (4.2)	1 (3.8)
≥12 months	28 (19.9)	4 (15.4)
Never	74 (52.5)	11 (42.3)
GP (for support with alcohol use), *n* (%)		
Past month	31 (22.0)	5 (19.2)
1 to <6 months	7 (5.0)	1 (3.8)
6–12 months	7 (5.0)	3 (11.5)
≥12 months	22 (15.6)	4 (15.4)
Never	74 (52.5)	13 (50.0)
Engaging in specialist alcohol treatment at baseline (within the past month), *n* (%)^c^	26 (18.4)	7 (26.9)
Index hospital episode		
Alcohol-attributable admission (narrow definition), *n* (%)	103 (73.0)	22 (84.6)
Emergency department attendance only, *n* (%)	40 (28.4)	5 (19.2)
Length of in-patient stay, median days (IQR)	7 (4–18.5)	7 (4–16.5)
First assessment of alcohol use in a secondary care setting, *n* (%)	60 (42.6)	7 (26.9)
Psychological distress		
HADS score, mean (s.d.)	21.4 (12.1)	22.0 (12.9)

IQR, Inter Quartile Range; AUDIT, Alcohol Use Disorder Identification Test; AUDIT-C, Alcohol Use Disorder Identification Test - Consumption questions; LDQ, Leeds Dependence Questionnaire; GP, General Practitioner.

a. Defined as >40 g (5 units) per day for women and >60 g (7.5 units) per day for men.

b. Includes specialist community or residential treatment service only.

c. ‘Specialist Alcohol treatment’ defined as attendance at a community/residential alcohol treatment service or alcohol-specific mutual aid group within the past month.

### Analysis

Analysis was conducted with SPSS (version 24 for Windows). Descriptive statistics were used to explore participant characteristics; paired sample *t-*tests assessed changes in participants’ mean level of alcohol consumption, psychological dependence, readiness to change and psychological distress, between baseline and 6-month follow-up. The number of participants who demonstrated favourable outcomes at 6 months was calculated. For the psychological dependence outcome, a change in LDQ score of ≥7 points was considered reliable, and follow-up scores had to fall to <7.3 for change to be considered clinically meaningful (see Supplementary Material, File 1, part S1 available at https://doi.org/10.1192/bjo.2020.138).

Data pertaining to participants’ experiences of attending hospital were extracted from transcribed interviews and analysed using the principles of thematic analysis.^28^ The initial coding scheme was developed by S.E.C., but themes were discussed within the research team to ensure comprehensive analysis. Nvivo (version 10 for Windows) software facilitated storage and coding of the data.

### Ethics

A lay expert panel and clinical staff involved in recruitment together provided feedback on the study protocol and documents before submitting for ethical review. The observational follow-up study was approved by the National Research Ethics Service's Northern Ireland Research Ethics Committee (reference 16/NI/0100), and qualitative interviews by the East of Scotland Research Ethics Committee (reference 17/ES/0005).

## Results

### Personal characteristics of study cohort at baseline

A total of 144 patients agreed to participate in the study (3 later withdrew consent). Table 2 outlines the characteristics of the overall cohort (*n* = 141) and the qualitative subsample (*n* = 26) at baseline. At baseline assessment, only 26 out of 141 (18.4%) participants reported they were already engaged with specialist community alcohol services.

Almost half the cohort (*n* = 60, 42.6%) reported that the index hospital episode marked the first discussion of their alcohol use in a secondary care setting. Most participants rated this discussion as either ‘very positive’ (*n* = 73, 51.8%) or ‘positive’ (*n* = 29, 20.6%), with only seven (5.0%) rating it as ‘negative’ (the remainder selected ‘neutral’). Participants who selected a ‘negative’ score most commonly reported doing so because they felt they needed in-patient medically assisted withdrawal from alcohol but were discharged from hospital without having undergone one. In terms of the nature of intervention offered by clinical staff, participants most commonly recalled receiving ‘advice’ about alcohol-related harms, ways to reduce consumption, medically assisted withdrawal from alcohol or relapse-prevention medication (*n* = 122, 86.5%). Less than half the cohort reported receiving information about available community alcohol treatment services (*n* = 58, 41.1%) and even fewer were directly referred (*n* = 35, 24.8%). Of those with no prior alcohol treatment history (*n* = 74), only 16 (21.6%) recalled being directly referred and 34 (45.9%) were ‘signposted’ to community treatment services.

### Outcomes at 6-month follow-up

At 6 months, 132 participants (93.6%) were successfully followed-up. Of the nine lost to follow-up, eight were uncontactable and one declined to be re-interviewed. Eleven participants (7.8%) died before the 6-month follow-up point, resulting in complete data-sets at both time points for 121 participants (85.8%). Those who died or were lost to follow-up (*n* = 20) were more likely to be men (90% *v.* 10%; *χ*^2^*=*4.11; *P* = 0.043), but they did not differ from the remaining cohort in other measured characteristics; longitudinal analyses are therefore based on observed cases (*n* = 121). Of the 11 participants who died, liver disease was the direct cause of death in 6 participants and was a contributory factor in 1 participant. ‘Alcohol Use Disorder’ was mentioned as a contributory factor for only two participants. None were engaging in specialist alcohol treatment at the time of baseline interview, and four had reported that their alcohol use had never been assessed in a secondary care setting before their index hospital episode.

During the 6 months between baseline and follow-up, 52 out of 121 (43%) participants reported accessing some form of support for their alcohol use (including online groups or talking to their general practitioner). Just under 40% (*n* = 48) accessed specialist alcohol treatment at least once during the follow-up period, of whom 17 (35%) did so for the first time and 39 (60.4%) were still engaging at the 6-month time point.

There were significant improvements at the group level across a range of outcomes, despite high levels of alcohol consumption and other measures of alcohol-related harm at the index episode (see Table 3). At follow-up, out of 121 participants, 92 (76%) reported drinking less during the past week than at baseline, 10 (8.3%) exhibited no change (5 continued to drink 7 days per week and 5 maintained abstinence) and 19 (15.7%) reported drinking more. A total of 55 participants (45.5%) reported no heavy drinking days during the past week, and 16 participants (13.2%) reported complete abstinence throughout the whole 6-month follow-up period.

**Table 3:** Change in drinking behaviour and other related variables between baseline (time 1) and follow-up (time 2) (*n* = 121)

Variables (range)	Mean score at baseline (s.d.)	Mean score at follow-up (s.d.)	Mean change (s.d.)	Mean change [95% CI]	*P*-value	Effect size (*d*)
Past-week unit consumption	139.67 (120.94)	63.78 (97.07)	−75.89 (131.38)	[−99.54 to −52.24]	**<0.001**	0.58
Past-week drinking days	5.64 (2.25)	3.34 (3.09)	−2.31 (3.26)	[−2.89 to −1.72]	**<0.001**	0.71
Past-week heavy drinking days	5.17 (2.59)	2.79 (3.10)	−2.38 (3.27)	[−2.97 to −1.79]	**<0.001**	0.73
AUD severity (AUDIT score, 0–40)	29.46 (7.40)	19.97 (11.51)	−9.50 (9.91)	[−11.28 to −7.71]	**<0.001**	0.96
Psychological dependence (LDQ score, 0–30)	18.45 (10.45)	10.93 (10.18)	−7.52 (10.05)	[−9.33 to −5.71]	**<0.001**	0.75
Problem Perception (SOCRATES score, 10–50)	37.74 (8.72)	31.41 (10.57)	−6.32 (9.11)	[−7.96 to −4.68]	**<0.001**	0.69
Taking Action (SOCRATES score, 6–30)	22.01 (6.71)	23.02 (6.99)	1.02 (8.93)	[−0.59 to 2.62]	0.213	0.11
Psychological distress (HADS score, 0–42)	21.46 (12.06)	16.52 (12.15)	−4.94 (10.97)	[−6.92 to −2.97]	**<0.001**	0.41

AUD, Alcohol use Disorder; AUDIT, Alcohol Use Disorder Identification Test; LDQ, Leeds Dependence Questionnaire; SOCRATES, Stages of Change Readiness and Treatment Eagerness Scale; HADS, Hospital Anxiety and Depression Scale.

There was a significant change on the ‘problem perception‘ subscale of the two-factor SOCRATES scale over the follow-up period (mean change −6.32, 95% CI −7.96 to −4.68; *P* < 0.001), which appeared to reflect the reduced drinking levels. The ‘taking action’ subscale showed a numerical, but non-significant increase over the follow-up period (mean change 1.02, 95% CI −0.59 to 2.62; *P* = 0.213), reflecting the ongoing effort to address levels of drinking. In terms of change in psychological dependence (as measured by the LDQ over the 6-month follow-up period), 60 out of 121 (49.6%) participants met criteria for reliable improvement, 56 (46.3%) made no reliable change and 5 (4.1%) increased their psychological dependence on alcohol. Of the 60 who reliably improved, 25 retained LDQ scores >7.3, indicating continued high psychological dependence on alcohol; of those making no reliable change (*n* = 56), 21 (37.5%) maintained low levels of psychological dependence from baseline to follow-up.

At follow-up, 58 out of 121 (47.9%) participants were below the threshold for clinically significant levels of dependence (i.e. LDQ score <7.3). However, of these individuals, 23 already had scored lower than this threshold at baseline (despite high levels of alcohol consumption), meaning it was not possible to make a clinically significant change. Therefore, a total of 35 out of 98 (35.7%) participants made a clinically significant change (moving from LDQ scores representing levels of psychological dependence seen in AUD clinical samples, to LDQ scores similar to those in a ‘well-functioning’ control group).

### Perception of the effect of hospital admission and alcohol interventions

Table 4 summarises the themes generated from analysis of qualitative interviews. Findings are described in detail in Supplementary Material, File 1, part S2, and include direct quotations from interviews (pseudonyms are used to protect participants’ identities), and a summary of the main themes is provided below.

**Table 4:** Themes and subthemes generated from analysis of qualitative interviews^a^

Main themes	Subthemes	Illustrative examples from patients^a^
Hospital as a ‘turning point’	Realising the effects of alcohol consumption	‘*When it became reality that I had the starts of scarring [of the liver] …it makes you think. I had no idea the amount I was drinking could do that…I mean that was a wakeup call. I don't want to die yet.*’ (Simon) ‘*Well I just couldn't believe it, you know? You just couldn't believe you could do that to yourself. It was a hell of a shock.*’ (Marie)
	The role of alcohol interventions	‘*It was brilliant, honestly it was like a magic wand had been waved over me…I was saved by the intensive care people doing the detox…I didn't know that it had been done, or how it had been done, or even what it is.*’ (Graham) ‘*They used to come and talk to me about alcohol…it didn't make me change my thinking or anything. It doesn't offend me that people might try to help…someone has got to do that in case people do want help…I guess it is [helpful] to some*’ (Robert) ‘*If it wasn't for these alcohol nurses referring me to [community treatment services],… I think there would be more people passed away unfortunately, and it would have been me, because I think I would still have been on the drink.*’ (Luke)
	Allowing time to reflect	‘*The first thing I thought of was my grandkids and my daughter, and my family, my sister. I have a family that care and love me. I felt sad that I had been so selfish; I was drinking because my parents died, but I'd leave my daughter in the same situation if I killed myself with the booze.*’ (Lynn) ‘It gave me time to reflect on my previous life, gave me a lot of time to stare at the walls. It gave me time to think about my life, times that have gone by, and I realised it's about time I knocked this s**t on the head.’ (Jack) ‘*Being in hospital and getting ‘detoxed’ just gives you space to think without the alcohol fog – you can't clearly think and decide what you want intoxicated.*’ (Joe)
From hospital to home	‘Back in the same old situation’	‘*I wasn't even thinking about drink. That was the last thing on my mind, but once I saw it, there it goes in my mind. I have just been detoxed and given 4 cans.*’ (Nathan, who had accepted accommodation in a ‘wet house’ to avoid being street homeless on discharge from hospital)
	Disjointed alcohol care pathways	‘*I was supposed to go down to [community treatment services], but they stitched me up. I did make the effort, but the guy was on annual leave. Don't see why they would have made an appointment for me if he was on annual leave. But no discredit to the man, he got mixed messages.*’ (Jack)
The stigma of AUD and hospital use	Self-stigma	*‘Several times I have been in hospital, and I hate myself for that because to me it feels like I am taking up a bed and I am wasting the doctor's time because there are people who are really sick out there….[for me] it was my fault, it was self-inflicted.*’ (Barbara)
	The ‘revolving door’	*‘Nobody in hospital has picked up on the fact, why? They just accept the fact that I am an ‘alcoholic’ and I am going to keep coming back…had they had found out why from admission 1, 2 or 3, I might not be sat here now with 40 plus admissions on my record.*’(Lee, army veteran with previously undiagnosed post traumatic stress disorder) ‘*It has become more of a “normal” thing for me, whereas to begin with you react with shock-horror, “what am I doing here?” Whereas I got to the stage where I was like, “oh look, I'm here again”.*’ (Donna)

AUD, Alcohol Use Disorder.

a. See Supplementary File 1, available at https://doi.org/10.1192/bjo.2020.138, for further details.

#### Hospital as a ‘turning point’

Hospital attendance often marked the first realisation for participants that alcohol intake had caused (in some cases serious and/or irreversible) physical harm. Many recalled feeling unwell before the index episode, but neglected to attend to their physical health, or drank more to mask pain. Several failed to recognise the association between physical ill health and alcohol use until this was made explicit during hospital attendance; an increased awareness of their morbidity and mortality often prompted participants to re-evaluate their alcohol use. Participants described the role staff played in this process: many were thankful for direct conversations about the seriousness of their alcohol intake, but some felt upfront conversations may evoke unnecessary fear, especially if they could not envisage how a change in their drinking would alter their poor prognosis. Participants generally agreed that hospital provided relief from the routine of everyday life, and afforded time to reflect on their relationship with alcohol and evaluate wider life goals.

#### From hospital to home

Participants often described a difficult transition back to their home environment following discharge. Despite high levels of readiness to change in hospital, an unsupportive home milieu increased risk of relapse back to heavy drinking, particularly for those with multiple and complex needs, including homelessness and mental illness. Restoration of physical health and faded memory of hospital attendance also increased vulnerability to relapse. Many participants voiced discontent at the lack of support available in the community and admitted to using hospital services for accessing help for their drinking. Disjointed pathways between hospital and community treatment were also said to undermine participants’ efforts to sustain change. A few examples of effective multi-agency working were given as facilitating favourable drinking outcomes and subsequent engagement with treatment.

#### The stigma of AUD and hospital use

Participants were generally positive about their treatment in hospital and welcomed discussions about their alcohol use. Staff were described as empathetic and supportive. However, many described ‘self-stigma’ for feeling a burden on NHS resources, and that their problematic alcohol use was the product of a moral failure. This was most often the case for participants who reported a history of multiple alcohol-related hospital attendances. Failure to understand the drivers of AUD and the resultant repeated hospital attendance seemed to serve to maintain them; this is highlighted in participants’ examples where person-centred holistic support was unavailable.

## Discussion

Much of the evidence about outcomes for patients with AUD is drawn from populations accessing specialist addiction services, although this constitutes only a minority of those with the condition.^8,29^ Although hospital episode statistics demonstrate an increase in patients with AUD presenting to hospital,^2^ less is known of the clinical characteristics, experiences, outcomes and potential treatment needs of this diverse patient group. This study highlights their high levels of morbidity and mortality, specifically, high levels of alcohol consumption (mean 140 UK alcohol units per week), severity of AUD (AUDIT score mean of 30/40), level of dependence (LDQ score of 18/30) and degree of psychological distress (HADS score of 21/42): and 11 out of 141 (8%) of participants died during the follow-up period.

The study findings provide evidence of the potential benefits of alcohol care teams, and illustrate some of the challenges in building integrated clinical pathways. Despite the high morbidity at baseline, the interventions facilitated positive change, with 55 out of 121 participants (45.5%) reporting no heavy drinking days in the week before follow-up, and 16 (13%) maintaining abstinence over the whole 6-month period since their hospital admission. In 102 out of 141 (72%) participants, being identified as having an AUD and receiving some targeted interventions to address this was welcome; and for 60 (43%) participants, it was the first time that this link between their health and alcohol use had been made clear, suggesting that opportunistic alcohol interventions can act as a ‘teachable moment’ for behaviour change.^30^

The study is limited by being undertaken in a convenience sample recruited from a single hospital. Southampton has the highest levels of all alcohol related admissions in the south-east region, and rates of alcohol-specific mortality (per 100 000) that are 50% higher than the national average (15.5 *v*. 10.6).^2^ Patients received a range of interventions before participation in the study, and all were identified as having an AUD and given feedback and onward referral when required. Therefore the study is not designed to test an intervention, but to explore the acceptability of alcohol-focussed interventions in patients presenting to acute hospitals with a range of health conditions.

A major strength of the study are that it explores the clinical characteristics and natural history of patients with AUD outside of specialist services, who may not be aware of the impact that alcohol is having on their health, or be seeking to change their behaviour. Additional strengths are the low level of attrition (6.4% at follow-up), and the use of mixed methods to explore the complexities and interactions between individual patient needs and expectations, and service provision (that would not be available using a single method).

The qualitative research findings indicate the importance of a wider pathway and coordinated services to support behaviour change and offer evidence-based relapse prevention interventions, especially for those with more complex mental health and social care needs.^5^ Recent guidance^31^ emphasises the importance of functioning pathways between the acute, community and mental health services, to prevent a loss of momentum around the motivation to change and support for comorbid conditions. But much remains uncertain, such as ascertaining the optimal measures for assessing clinical outcomes, identifying the ‘active ingredients’ of successful interventions and establishing potential predictors of response. The potential impact of alcohol interventions on outcomes when delivered to patients admitted to mental health services also requires further exploration.

## Data Availability

Research data associated with the quantitative analyses can be accessed via the University of Southampton repository (doi: 10.5258/SOTON/D0689).

## Supplementary material

For supplementary material accompanying this paper visit http://dx.doi.org/10.1192/bjo.2020.138. click here to view supplementary material
