# Supplementary material for: Course and outcome of patients with alcohol use disorders following an alcohol intervention during hospital attendance: mixed method study
Source: BJPsych Open. 2020 Dec 2;7(1):e6. doi: 10.1192/bjo.2020.138 (PMC7791568; doi:10.1192/bjo.2020.138)
Supplement: Supplementary file 1 [file S2056472420001386sup001.docx]

**S1: Reliable and clinically significant change calculation**

The formula to calculate reliable change proposed by Jacobson and Truax (1991) is:

$$\boldsymbol{Reliable change=}\frac{\boldsymbol{x}_{\boldsymbol{2}}\boldsymbol{-}\boldsymbol{x}_{\boldsymbol{1}}}{\boldsymbol{S}_{\boldsymbol{diff}}}$$

­­­­­­­­­­­­­­­­

where x_1_ represents a participant’s pre-test score, x_2_ represents a participant’s post-test score and S_diff_ is the standard error of the difference between the scores. S_diff_ can be calculated from the standard error of measurement (S_E_) according to the formula:

$$\boldsymbol{S}_{\boldsymbol{diff}}\boldsymbol{=}\sqrt{\boldsymbol{2(}\boldsymbol{S}_{\boldsymbol{E}}\boldsymbol{)}^{\boldsymbol{2}}}$$

Standard error is calculated as:

$$\boldsymbol{S}_{\boldsymbol{E}}\boldsymbol{=}\sqrt[\boldsymbol{S}_{\boldsymbol{1}}]{\boldsymbol{1-}\boldsymbol{r}_{\boldsymbol{xx}}}$$

where r_xx_ is the test re-test reliability of the measure (reported as 0.95 in the LDQ validation study (Raistrick et al., 1994), and S_1_ is the standard deviation for the mean total LDQ score for the cohort at baseline, 10.29). Therefore, S_E_ was calculated to be 2.30, and S_diff_ to be 3.25. A reliable change score could then be calculated for each participant; Jacobson and Truax (1991) propose that a score ≥1.96 is unlikely to have occurred by chance (this translates to a minimum score change of 7 points on the LDQ in the current study).

Next, to establish which participants had demonstrated a clinically significant change, a cut-off LDQ score was calculated according to the following formula:

$$\boldsymbol{Cut off score=}\frac{\left( \boldsymbol{SD func*Mean dys} \right)\boldsymbol{+(SD dys*Mean func)}}{\boldsymbol{(SD dys+SD func)}}$$

*func = ‘functional’ normative population; dys = ‘dysfunctional’ clinical population*

Normative data were based on the sample described by Raistrick & Tober (2019; <https://www.result4addiction.net/ldq>); n=870 opportunistic NHS and local university sample (male n=220):

$$\boldsymbol{Cut off score=}\frac{\left( \boldsymbol{4.15*18.43} \right)\boldsymbol{+(10.29*2.77)}}{\boldsymbol{(10.29+4.15)}}\boldsymbol{=7.3}$$

Participants whose LDQ scores reduced to below the cut-off at follow-up (i.e. 7.3^[[1]](#footnote-2)^) were considered to have made a clinically significant change as their level of dependence moved closer to that found within a well-functioning population.

**S2: Extended qualitative findings**

1. **Hospital as a “turning point”**

***1.1 Realising the effects of alcohol consumption***

Several participants reported that hospital attendance marked the first realisation that alcohol intake had caused (in some cases serious and/or irreversible) physical harm:

*“When it became reality that I had the starts of scarring [of the liver] …it makes you think. I had no idea the amount I was drinking could do that…I mean that was a wakeup call. I don’t want to die yet.”* (Simon)

Although many participants could recall feeling unwell prior to hospital attendance, they often ignored earlier signs of ill-health or drank more to mask them. Several said they *“didn’t really associate [physical symptoms] with alcohol at the time”* (Joe); it was only with hindsight and knowledge learned during their hospital stay(s) that the effects of alcohol on the body were recognised. Joe continued: *“It was only really then [in hospital] that I knew there was a connection with my drinking, it was explained to me then.”*

***1.2 The role of alcohol interventions***

Admission to hospital was often described as a *“relief”* in that it provided access to support and intervention from healthcare professionals, sometimes for the first time. Being in hospital often increased awareness of mortality, which served as a *“wake-up call”* and prompted re-evaluation of alcohol use. Many participants recalled conversations with medical staff during their hospital admission about the seriousness of their physical ill-health and the impact of continued drinking:

*“The best thing a consultant ever said to me, he said: ‘If you go out of here and drink, you’ll come back into hospital and out again in a coffin’…the scare tactic worked.”* (Luke)

However, if participants could not envisage that a change in their drinking would alter the course of poor health, provision of feedback about health status sometimes had the opposite effect: *“if I was going to die, I might as well make it happen quicker to save the agony”* (Jack). Nevertheless, many participants felt healthcare professionals had a duty to *“tell it as it is”,* and agreed with Clive who said, *“I absolutely do not feel medics should tip-toe around…out of fear of offending the person”.* In fact, several said they believed upfront conversations were the most effective intervention for changing behaviour. Although this direct approach may not be universally effective, participants were in overwhelming agreement that it *“doesn’t hurt anybody to have a chat”* (Glen), and were receptive to interventions in hospital for people with AUD.

***1.3 Allowing time to reflect***

Participants frequently said hospital admission marked a “turning point” because it afforded them time to reflect on their relationship with alcohol and evaluate wider life goals. Respite from the daily routine of alcohol consumption to allow *“thinking space”* (Simon) was highlighted as a positive by-product of the hospital episode more often than restoration of physical health, which for some participants appeared to be an afterthought: *“the time in there…I didn’t have to think about anything or worry about anything apart from my drinking, oh and my health obviously”* (Howard).

The psychological break that hospital admission provided often symbolised the start of a new life chapter for many participants, even if they resumed drinking post-discharge. Jack’s reference to his “previous life” before his hospital admission exemplifies this:

*“It gave me time to reflect on my previous life, gave me a lot of time to stare at the walls. It gave me time to think about my life, times that have gone by, and I realised it’s about time I knocked this s**t on the head.”*

1. **From hospital to home**

***2.1 “Back in the same old situation”***

Even despite high levels of readiness to change in hospital, several participants found difficulty in transitioning back to their home environment following discharge:

*“You say all these things to yourself in hospital about how you’re not going to drink again but when I got home, I started again…you are back in the same old situation and you go back to your old ways.” (Marie)*

The transition was particularly challenging for participants with multiple and complex needs, such as homelessness and mental illness. Nathan, for example, accepted residence at a ‘wet house’ which operated a scheduled drinking programme, to avoid rough sleeping:

*“I wasn’t even thinking about drink. That was the last thing on my mind, but once I saw it, there it goes in my mind. I have just been detoxed and given 4 cans.”*

Some participants explained that as their memory of hospital admission faded, so did their recognition of alcohol as a problem:

*“Initially [hospital admission], it made me think, ‘curtail it, slow it down a bit…[but] after about a week, how shall I put it? Relapse – ‘oh I’m better now’.”* (Trevor)

Extending Trevor’s point, several participants explained that improvements in physical health made less salient the need to maintain abstinence. For example, Daniel, who was interviewed during a subsequent alcohol-related hospital admission, said:

*“Two or three days ago I felt like death, I really felt like I was on my way out. Now I feel great. I have already lost the feeling and memory of how bad it can be, you know this is only two days ago.”*

***2.2 Disjointed alcohol care pathways***

Several participants described using hospital services as a means of accessing support for their drinking, as other treatment options were inadequate or unavailable. For example, Joe explained that he was placed on a waiting list for an appointment at his local community alcohol service but *“needed something immediately”* and so presented to hospital:

*“I knew I needed help. So, I almost force-drank myself into hospital. I went up to A&E, they discharged me within 2 hours. So, I went home, drank more, and then went back to hospital and they kept me in for a couple of nights…I just thought, ‘I need to make myself really ill. I know I need help, but I just seemed to have been told to go home’. Right ok, I will just try harder.”*

However, disjointed pathways between hospital and community treatment were said to undermine participants’ efforts to sustain change. Jack, for example, was given an appointment to attend community treatment services by hospital staff but was turned away:

*“I was supposed to go down to [community treatment services], but they stitched me up. I did make the effort, but the guy was on annual leave. Don’t see why they would have made an appointment for me if he was on annual leave.”*

Examples of effective multi-agency working, reinforce its importance for favourable drinking outcomes and subsequent treatment utilisation, even if only for individuals with severe AUD who often require ongoing support: *“If it wasn’t for these alcohol nurses referring me to [community treatment services], I would still have been on the drink.”* (Luke)

1. **The stigma of AUD and hospital use**

***3.1 Self-stigma***

Feedback about staff was generally positive, for example:

*“The respect with which each individual is treated is second to none, it really is. It made no difference to them that I needed some treatment for alcohol.”* (Clive)

Participants were much more likely to describe ‘self-stigma’ and many expressed concerns about *“wasting NHS resources”,* especially if they reported several alcohol-related hospital attendances. Self-defamatory statements often stemmed from assumptions about AUD being the product of “moral failing”:

*“Several times I have been in hospital, and I hate myself for that because to me it feels like I am taking up a bed and I am wasting the doctor’s time because there are people who are really sick out there….[for me] it was my fault, it was self-inflicted.”* (Barbara)

***3.2 The “revolving door”***

Individuals were most likely to describe negative experiences with staff if they had a history of multiple alcohol-related hospital attendances (*“I am what you would call a ‘revolving door’ patient”*, Daniel):

*“How many times are you going to go into hospital, how many times are people going to have to help you? Eventually they will go, ‘oh it’s that f*****g drunk again, let’s get rid of him’.”* (Howard)

Participant examples where person-centred holistic support was unavailable, highlight how failure to understand the drivers of alcohol use disorders and repeated hospital attendance can serve to maintain them. Lee, an army Veteran, for example, was only diagnosed with posttraumatic stress disorder (PTSD) and referred to specialist support services after his new manager *“noticed something [was] wrong”*:

*“Nobody in hospital has picked up on the fact, why? They just accept the fact that I am an ‘alcoholic’ and I am going to keep coming back…had they had found out why from admission 1, 2 or 3, I might not be sat here now with 40 plus admissions on my record.”*

1. The same cut-off score was used for males and females as there was no difference between their LDQ scores at baseline (*M =* 18.46 and 18.34 respectively); t(139) = -.062, p = .951. [↑](#footnote-ref-2)
